# Expanding Access to Oral Preexposure Prophylaxis for People Who Inject Drugs in Bayelsa and Niger States, Nigeria

**DOI:** 10.9745/GHSP-D-22-00370

**Published:** 2023-02-28

**Authors:** Lirica Nishimoto, Patrick Ikani, Joseph Achanya, Adekunle Idowu, Adaobi Lisa Olisa, Christa Fischer Walker, Amy Gottlieb, Christopher Akolo

**Affiliations:** a FHI 360, New York, NY, USA.; b FHI 360, Abuja, Nigeria.; c Achieving Health Nigeria Initiative, Abuja, Nigeria.; d FHI 360, Washington, DC, USA.

## Abstract

**Introduction:**

While oral preexposure prophylaxis (PrEP) is recommended for all individuals with substantial risk of acquiring HIV, people who inject drugs (PWID) have not been prioritized for oral PrEP services in most settings. The Meeting Targets and Maintaining Epidemic Control (EpiC) project implemented a comprehensive community-based program to increase access to and uptake of oral PrEP services among PWID in Bayelsa and Niger States, Nigeria.

**Program Description:**

EpiC established partnerships with community-based organizations working with the PWID community, identified hotspots (common locations for socialization and drug use), and engaged PWID in tailoring oral PrEP services. The HIV prevention package provided in hotspots and drop-in centers included HIV testing, oral PrEP, postexposure prophylaxis, sexually transmitted infection screening and management, condoms and lubricants, PWID-specific risk reduction counseling, and referrals to other support programs. At drop-in centers, paralegal and psychological services were also provided. Peer outreach workers led outreach efforts. All PWID who tested HIV negative were eligible for oral PrEP screening. National oral PrEP eligibility criteria included: no suspicion of acute HIV infection, absence of proteinuria, and willingness to use oral PrEP as prescribed. We used descriptive analysis of routine programmatic data to illustrate program outcomes.

**Results:**

From January 2020 to September 2021, 13,286 HIV tests were completed for PWID. Of these, 12,111 (91.16%) had negative results. Among those testing negative, 8,190 (67.62%) were screened for oral PrEP eligibility, and 2,661 (32.49%) were eligible. A total of 2,659 PWID (100% [312/312] among eligible females; 99.91% [2,347/2,349] among eligible males) initiated oral PrEP.

**Lessons Learned:**

Provision of oral PrEP services to PWID in Nigeria is feasible through a peer-led, differentiated, and holistic approach that addresses structural barriers among the PWID community. Greater efforts are needed to understand the needs and barriers to better tailor HIV services to reach females and other subpopulations in this community that are harder to reach.

## Abstract (precis)

Optimizing the impact of oral preexposure prophylaxis HIV interventions for people who inject drugs requires increasing access to services through different service delivery models and creating an environment where community members feel safe and motivated to access services.

## Teaser

**Key Findings:**

- A community-based distribution program addressed barriers to access and uptake of oral preexposure prophylaxis (PrEP) among people who inject drugs (PWID) by providing a comprehensive package of services, including HIV testing, sexually transmitted infection screening, oral PrEP, risk reduction counseling, and other health and support services.
- Using differentiated service delivery models, providing person-centered holistic HIV prevention services that address the broad range of needs of PWID, and delivering services in an environment where PWID feel safe may increase access to and acceptance of services.

**Key Implications:**

- In addition to scale-up of oral PrEP services, further program evaluation is needed to inform and improve program interventions to reach PWID with HIV prevention services and address PWID-specific barriers to uptake of services.
- Better tailoring of oral PrEP services for women who inject drugs—who have a higher prevalence of HIV—and older PWID, who may be harder to reach, is needed.

## INTRODUCTION

In 2015, the World Health Organization recommended preexposure prophylaxis (PrEP) as an HIV prevention tool for populations with substantial risk of acquiring HIV, including people who inject drugs (PWID).^1^ In 2016, the Joint United Nations Programme on HIV/AIDS (UNAIDS) set a goal to ensure comprehensive HIV prevention coverage, including oral PrEP, for 90% of people at risk of HIV infection, with a focus on key populations (KPs), by 2020.^2^ However, only 78 countries currently offer oral PrEP in some form, and the global estimated percentage of PWID at risk of HIV infection on oral PrEP is far below the 2016 target.^3^^,^^4^

PWID are 1 of the 5 KP groups that carry a high burden of HIV globally, as identified by the World Health Organization.^5^ Among the estimated 12.7 million PWID worldwide in 2014, 13%, or 1.7 million, were living with HIV.^6^ In 2021, it was estimated that PWID had 35 times higher risk of acquiring HIV compared to the general adult population.^7^ KPs and their sexual partners accounted for 70% of new HIV infections,^7^ and specifically, it was estimated that PWID accounted for 10% of new HIV infections globally in 2019.^8^ While needle exchange programs have improved HIV prevention outcomes among PWID, in many settings, political and structural barriers prevent access to these programs. PWID also engage in high-risk sexual behaviors, and the HIV epidemic continues to grow.^9^^,^^10^ Therefore, scale-up of HIV prevention interventions, including oral PrEP, for PWID is necessary to reach epidemic control.

Scale-up of HIV prevention interventions, including oral PrEP, for PWID is necessary to reach epidemic control.

A randomized controlled trial conducted in Thailand found that oral PrEP use effectively and safely reduced HIV incidence among PWID,^11^ and studies in Ukraine, Canada, and India have found that oral PrEP is acceptable among PWID (85.0%, 35.4%, and 52.4%, respectively).^12^^–^^14^ However, despite willingness among PWID to use oral PrEP, uptake can be a challenge.^15^

Globally, PWID have not been prioritized for oral PrEP services in part due to criminalization of and stigma associated with injection drug use in many countries.^16^ Among 59 countries with data available on the national oral PrEP target populations, 7 specified PWID, 11 mentioned high-risk populations or KPs but did not specify PWID, and the remaining 41 did not mention PWID or any population category inclusive of PWID.^3^ Nigeria adopted an oral PrEP policy in 2016 recommending oral PrEP as part of combination prevention for individuals at high risk of acquiring HIV, with emphasis on serodifferent couples and KP individuals.^17^

The 2021 estimate of PWID population size in Nigeria was 326,100, with an HIV prevalence of 10.9%, which was higher than the prevalence in the general population of those aged 15–49 years (1.3%). In 2021, an estimated 37.2% of PWID were aware of their HIV status, 46.5% used condoms, 35.9% conducted safe injecting practices, and 25% of those who tested positive were on antiretroviral therapy (ART).^18^ PWID in Nigeria account for up to 9% of new HIV infections.^19^

Globally, it is estimated that 1 in 5 PWID are women.^20^ In Nigeria, 17% of PWID are estimated to be women.^21^ However, the disproportionate burden of HIV on women in Nigeria is also reflected among PWID, with women who inject drugs having a higher HIV prevalence (13.9%) than men who inject drugs (2.6%).^19^ A 2018 survey found that female PWID were more likely to report sharing needles and syringes compared to male PWID, and female high-risk drug users, including PWID, were more likely to engage in sexual HIV risk behavior than male PWID, increasing their exposure to HIV transmission.^21^ Less than half of all high-risk drug users, and an even lower proportion of PWID, were estimated to access HIV testing services (HTS).^18^^,^^21^ Similarly, access to oral PrEP services in Nigeria remains low, with an estimated 276,761 people at risk of HIV initiated on oral PrEP.^22^

While injection drug and needle-sharing behaviors are frequently cited as factors contributing to the heightened risk of HIV transmission among PWID, their sexual HIV risk behavior, including unprotected sex and engaging in commercial or transactional sex for drugs, cannot be ignored.^9^ An assessment conducted in Nigeria found that among KPs assessed (female sex workers [FSW], men who have sex with men [MSM], and PWID), PWID were significantly more likely to engage in unprotected sex and to allow sex when the partner refuses to use a condom, compounding their risk of transmission.^23^ A separate assessment found that 44% and 60% of high-risk drug users, including PWID, have received drugs or money to have sex, respectively. Injection drug behavior was also prevalent among FSWs and MSM (25% of the FSW respondents and 29.4% of MSM respondents).^21^ Additionally, drug use in Nigeria is illegal. A 2018 survey found that high-risk drug users, including PWID, in Nigeria have been arrested an average of 3 times,^21^ highlighting the likelihood of arrest among this population.

In this context, the Meeting Targets and Maintaining Epidemic Control (EpiC) project implemented oral PrEP services—specifically, the provision of Truvada as it was the only PrEP option available in Nigeria at the time—as part of a comprehensive package of services for PWID in Bayelsa and Niger states, Nigeria. We describe the multipronged approach of the project and the population- and context-specific adaptations, lessons learned using routinely collected PEPFAR indicator data, and implications for future scale-up of oral PrEP programs for PWID in Nigeria.

## PROGRAM DESCRIPTION

### Overview

EpiC is an FHI 360–led global project funded by the U.S. President’s Emergency Plan for AIDS Relief (PEPFAR) and U.S. Agency for International Development (USAID). With support from the Key Populations Investment Fund, the project in Nigeria provided comprehensive HIV prevention, care, and treatment services to KPs, including FSW, MSM, transgender persons, prison inmates, and PWID in Bayelsa and Niger states. PEPFAR, USAID, and the National Agency for the Control of AIDS (NACA) identified Bayelsa and Niger States as priority states for EpiC/Key Populations Investment Fund for additional investment and focused programming based on nationally triangulated HIV prevalence data and the lack of existing HIV programming for KPs. The program expanded HIV services to hard-to-reach populations by working with community-based organizations (CBOs) and local opinion leaders. The PWID program was embedded within the overall HIV program for KPs but with adaptations to meet the specific needs of the PWID community.

### Scoping Visit

At the start of the project, a scoping visit led by the EpiC team’s senior program and technical staff in close collaboration with other stakeholders, primarily the local CBOs and the State AIDS and STD Control Programme, was conducted in the 2 states to: (1) identify potential local partners with technical and organizational capacity to deliver services to KPs, including PWID; (2) conduct advocacy to stakeholders and sensitize them on the upcoming program; (3) understand the existing services for KPs; and (4) assess opportunities available for provision of the comprehensive package of services to KPs. The scoping visit provided an opportunity to identify potential local partners to collaborate with in delivering HIV services to PWID and to establish a baseline of the PWID population size. The scoping visit was followed by partnering with CBOs based on their experience working with and their knowledge of public health programming for PWID. Two CBOs, Kindling Hope Across Nations Initiative in Bayelsa State and Centre for Communication and Reproductive Health Services in Niger State, were selected through a competitive process. Although there were no HIV programs specifically for PWID before the commencement of the EpiC-supported program, these organizations were already actively engaging PWID community members as peer providers and ad hoc program staff. Therefore, the EpiC program was built on the preexisting community reach provided by these two CBOs.

### Mapping Exercise

With no baseline data for PWID in Bayelsa and Niger states, the EpiC project conducted a hotspot mapping exercise, engaging the PWID community and its leaders through Kindling Hope Across Nations Initiative and Centre for Communication and Reproductive Health Services, from January through March 2020. The mapping exercise was conducted to identify hotspots where PWID frequently socialize with each other and inject drugs. Through this exercise, a total of 209 hotspots (154 active hotspots in Bayelsa and 55 in Niger State, respectively) were identified, and the PWID population was estimated to be 6,032 individuals (2,134 in Bayelsa and 3,898 in Niger). In Bayelsa State, PWID live near creeks and coastal communities where theft of crude oil occurs. Across Niger State, injection drug use is prevalent in communities where mining and quarrying occur. PWID communities in both states have an unofficial hierarchical structure with community leaders who serve as guardians of the PWID community.

In both states, PWID face barriers to accessing care, including lack of awareness of available services, fear of arrest when accessing care, fear of stigma and discrimination at health facilities, and the inability to afford services.

### Oral PrEP Program to Reach PWID

The EpiC PrEP program for PWID used a multipronged approach to reach the PWID community and to improve the PWID community's access to and uptake of oral PrEP by leveraging existing community structures and addressing known barriers to access experienced by this population.^24^

The EpiC program used a multipronged approach to improve the PWID community's access to and uptake of oral PrEP by leveraging existing community structures and addressing known barriers to access.

#### Community Engagement

The mapping exercise engaged PWID community leaders and members early in the project cycle. The PWID community contacts were sensitized about the EpiC project; its provision of HIV/AIDS prevention, care, and treatment services; and benefits for the PWID community. The community actively participated in intervention planning, outreach activities, and nomination of PWID community members as peer outreach workers. Peer outreach workers also participated in microplanning, where they identified and mapped PWID individuals most at risk to prioritize them for outreach for oral PrEP service provision. Monthly meetings were held at the CBOs with all peer outreach workers to review program data, discuss program challenges, and offer solutions to emerging issues that might impede program implementation.

#### Holistic PWID-Tailored Interventions

Although general HIV services that reached some individuals in the PWID community started in December 2019, PWID-tailored interventions, including oral PrEP services, were not initiated until February 2020, when mapping data became available. Oral PrEP was provided as part of the combination package of prevention services. The HIV prevention package for PWID targeted both prevention of sexual and intravenous transmission of HIV and, in addition to oral PrEP, included HTS, sexually transmitted infection symptomatic screening and management, condoms, and risk reduction counseling. Risk reduction counseling included discussion on strategies for the prevention of HIV transmission via sex and needle sharing, including oral PrEP, postexposure prophylaxis, use of condoms and lubricants, and limiting needle sharing. The CBOs also collaborated with other service providers in neighboring states identified during the scoping visit and referred clients to the additional services, including economic empowerment programs, drug rehabilitation homes, and harm reduction programs. Needle exchange and other harm reduction services were not available within the project.

#### Differentiated Service Delivery Models

EpiC implemented differentiated and integrated oral PrEP service delivery models to reduce barriers and increase access. For example, HTS, ART, and oral PrEP were made available through a range of service delivery modalities, including home delivery by peers, courier service delivery, community pharmacies, support groups, and after-hour service delivery through community outreach led by peer outreach workers and community ART (CART) teams at hotspots. Commodities were dispensed at all the access points through trained peer outreach workers and service providers as well as through courier services after a virtual consultation with clients. Additionally, the 2 CBOs had drop-in centers (DICs) which served as a safe space and access points for the full HIV prevention package of services, ART initiation and refills, viral load specimen collection, clinical and mental health screenings, paralegal support, psychological support, hepatitis B and C screenings using rapid test kits, and referrals to public health facilities for medical treatment, as needed. The locations of the 2 DICs were chosen in consultation with the PWID community to ensure easy access and that services can be accessed in a stigma-free community service delivery model. Oral PrEP commodities and other services were paid for by PEPFAR/USAID and provided at no cost to the beneficiaries while some clients received transportation reimbursement to access services. Table 1 outlines the roles of each community service provider and the services provided.

**TABLE 1.** Roles and Activities Led by Community Service Providers During Oral PrEP Service Delivery Program for PWID, Bayelsa and Niger States, Nigeria

**Community Service Provider**	**No. Engaged**	**Role and Services Provided**
PWID peer outreach workers^a^	10	Provided basic information about HIV prevention, oral PrEP screening, counseling, home delivery of oral PrEP, and linkage and referrals to DICs for additional services Led outreach, demand creation, trust-building activities at hotspots, education, and discussions on HIV/STI prevention and treatment at the community level Provided incentives (e.g., mosquito nets, condoms, face masks) to PWID for attending health education sessions
CART team members^b^	10	Provided outreach to PWID hotspots to counsel, test, initiate ART, provide oral PrEP and other prevention services, and facilitate referrals for other services

Abbreviations: ART, antiretroviral therapy; CART, community antiretroviral therapy; DIC, drop-in center; M&E, monitoring and evaluation; PrEP, preexposure prophylaxis; PWID, people who inject drugs; STI, sexually transmitted infection.

a Members of the PWID community.

b Including pharmacist, clinician, lab technician, social worker, and M&E staff.

HTS, ART, and oral PrEP were available through home delivery by peers, courier service delivery, community pharmacies, support groups, and after-hour service delivery through community outreach.

#### Peer-Led Service Delivery

The peer outreach workers, CART team members, and other service providers who have been previously trained to provide KP-friendly services were trained on using available oral PrEP training materials adapted to address PWID specific issues. The PWID clients who tested HIV negative through any service delivery channel were counseled and screened for oral PrEP eligibility. Clients were eligible to initiate oral PrEP if they tested negative, had no suspicion of acute HIV infection, had no evidence of proteinuria, and were willing to use oral PrEP as prescribed. Expanding access to and coverage of HTS provided a large pool of HIV-negative PWID clients eligible for oral PrEP screening. Drug barons, who are the leaders of small drug distribution circles and are usually the main source of the drugs distributed to PWID, were identified by the peer outreach workers and sensitized on oral PrEP, and they leveraged their influence as PWID community leaders to encourage many PWID to uptake oral PrEP.

As part of adaptations to the COVID-19 pandemic and its associated lockdowns, EpiC launched virtual case management utilizing audio and video calls, text messaging, and WhatsApp groups to increase reach by virtual outreach workers, provide adherence support, and broaden acceptance of oral PrEP within the PWID community.

Given the criminalization of drug use in Nigeria, the EpiC project worked to create an enabling environment for PWID to access care with minimal interference and fear of arrest, including through advocating with the National Drug Law Enforcement Agency (NDLEA) to avoid arresting the program’s clients. Table 2 describes the timeline of activities and interventions implemented to create an enabling environment for the PWID community to access services and for the supported CBOs to further build trust within the PWID community.

**TABLE 2.** Timeline of Key Activities Implemented During Oral PrEP Service Delivery Program for PWID, Bayelsa and Niger States, Nigeria

**Date**	**Activity**	**Description/Stakeholders Involved**
December 2019	Commenced HIV program implementation	Initiated HIV services, except oral PrEP, through PWID-focused CBOs to engage PWID PWID engaged during this initial outreach effort helped to identify PWID community leaders who were engaged in the subsequent mapping exercise for oral PrEP programming. Peer outreach workers were also identified through this initial outreach effort allowing for more peer-led efforts for oral PrEP demand creation.
January–March 2020	Conducted mapping and size estimation	Mapped PWID communities in both states to identify PWID hotspots where oral PrEP demand creation and services can be provided, estimated the size of the population, identified available services, and planned for scale-up of oral PrEP services
February 2020	Initiated prevention services	Initiated oral PrEP services tailored to PWID in both states
March–October 2020	Initiated strategies to continue services through COVID-19 lockdown	Adjusted oral PrEP service outreach hours among peer outreach workers to accommodate curfew time and lockdown periods Provided virtual case management and telemedicine for PWID clients for continued oral PrEP demand creation and adherence support Provided virtual supportive supervision, mentoring, and capacity building for staff of CBOs and for peer outreach workers providing oral PrEP Negotiated with the government to permit movement for HIV service and commodity provision, including oral PrEP, during periods of lockdown
March–April 2020	Oriented stakeholders at monthly interface meetings	Included 72 CBO staff, the PWID community, the Niger and Bayelsa state governments, the Nigerian Police Force, NSCDC, NDLEA, and community vigilante teams Introduced the EpiC program to all stakeholders Oriented stakeholders on importance of access to health care and HIV prevention, such as oral PrEP, for PWID without fear of being arrested Educated the PWID community about availability of an arrest-free rehabilitation program provided by the NDLEA to address some fear of arrest among the PWID community that prevent them from seeking and accessing health services, including oral PrEP
March–April 2020	Provided trainings on human rights and stigma and discrimination	Included 104 CBO staff, health care providers, paralegal security personnel, community vigilante teams, NSCDC, SACA, and SASCP Created awareness on human rights and fostered collaboration between the community and law enforcement agencies to foster an enabling environment where individuals of the PWID community feel safe in accessing health services, such as oral PrEP Educated participants on the barriers to providing quality and comprehensive HIV services to PWID Brought to attention the stigma and discrimination faced by the PWID population as a barrier to them accessing necessary health services, including oral PrEP, and the efforts needed to address this
July 2020	Engaged oral PrEP champions	Included 6 oral PrEP champions (members of the PWID community who were on oral PrEP) responsible for providing peers with information about oral PrEP and supporting adherence and continuation Raised awareness of oral PrEP and its benefits among PWID Encouraged PWID with ongoing risk to continue oral PrEP usage
July–August 2020	Held community dialogues	Held 2 discussions with 8 PWID community leaders, 6 security personnel, and government agency representatives to increase awareness of the importance of ensuring PWID have access to comprehensive HIV care and to increase demand for health services and oral PrEP among PWID
July–August 2020	Sensitized KP community on oral PrEP	2 meetings included 8 KP community leaders, among them PWID community leaders Sensitized KP leaders on oral PrEP benefits for PWID to encourage their support in demand creation and promotion among the PWID community
August 2020	Conducted oral PrEP drive	Worked with members of the PWID community and peer outreach workers to conduct periodic surges of outreach activities to increase oral PrEP screening and uptake among PWID
August 2020	Trained peer outreach workers in motivational counseling	Included 10 PWID peer outreach workers in training on how to motivate PWID to take oral PrEP and access other HIV prevention, care, and treatment services Improved counseling and communication skills among all peer outreach workers
November 2020	Trained PWID on as paralegals	Trained 30 PWID as paralegals to interface with security agencies in cases of harassment or discrimination against PWID community members to improve confidence among the PWID community in their security when accessing health services, including oral PrEP

Abbreviations: CBO, community-based organization; EpiC, Meeting Targets and Maintaining Epidemic Control; KP, key population; NDLEA, National Drug Law Enforcement Agency; NSCDC, Nigerian Security and Civil Defense Corps; PrEP, preexposure prophylaxis; PWID, people who inject drugs; SACA, State Agencies for the Control of AIDS; SASCP, State AIDS and STD Control Programme.

### Ethical Approval

All clients were educated on and informed about oral PrEP before it was offered. All clients who enrolled on oral PrEP gave their informed consent. Those who did not agree to use oral PrEP as prescribed were not eligible for and did not initiate oral PrEP. All data included in this article were extracted from the routine project performance reports. The authors did not have access to individual-level data.

## RESULTS

Between January 2020 and September 2021, a total of 13,286 HIV tests (both first-time and repeat testing) were conducted among PWID in the 2 states. Among the number of HIV tests conducted, 12,111 (91.16%) had negative results. Among the negative test results, 8,190 (67.62%) were followed by oral PrEP eligibility screening. Among those eligible (2,661 [32.49%]), nearly all (2,659 [99.92%]) were initiated on oral PrEP.

Due to the oral PrEP stock shortages during the first 7 months of implementation, we were limited in the number of HIV-negative PWID clients to whom we could offer screening and oral PrEP. After July 2020, the proportion of those screened among those who tested HIV negative improved (49.43% in January–July 2020 vs. 87.03% in August 2020–September 2021). Of note, a proportion of HIV tests were conducted among PWID clients who were already initiated on oral PrEP through the program and were accessing HTS prior to their oral PrEP refill to confirm their HIV seronegative status. Hence, some HIV-negative test results were not followed by oral PrEP screening.

A higher volume of male than female PWID were reached (Figure) (87.57% of PWID who tested for HIV were male and 12.43% were female), which reflects the PWID population demographic. Differences were observed between female and male PWID across the cascade of services. A smaller proportion of female PWID tested HIV negative (86.92% female vs. 91.76% male), which is consistent with the NACA-reported HIV prevalence (13.9% among female vs. 2.6% among male).^18^ While a lower proportion of females than males was screened for oral PrEP (57.17% vs. 69.03%), a higher proportion of females was eligible for oral PrEP (38.00% vs. 31.88%). Initiation was similar between sexes (100% [312/312] female vs. 99.91% [2,347/2,349] male).

**FIGURE.**
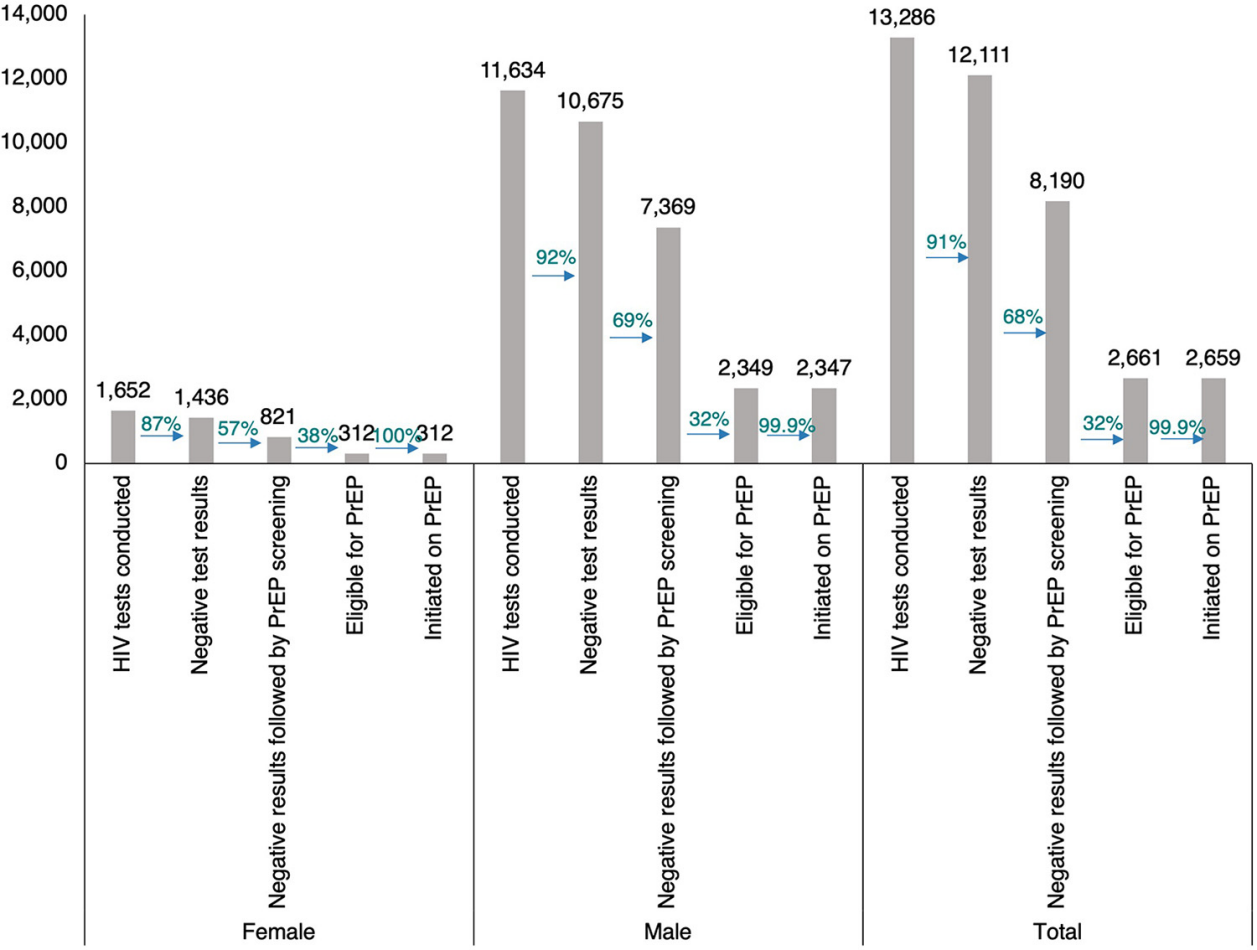
Oral PrEP Cascade by Sex, Bayelsa and Niger States, Nigeria, February 2020–September 2021 Abbreviation: PrEP, preexposure prophylaxis.

Overall, most (80.99%) PWID reached by the program were aged 20–34 years; only 15.18% were aged 35 years or older and 3.80% were aged under 20 years.

## LESSONS LEARNED

### Build Trust Through Community Input and Engagement

The multipronged approach to oral PrEP service delivery among the PWID population facilitated trust with the PWID community and PWID leaders, allowing program implementers and service providers to bring the services to PWID. The trust with the PWID community was built from the start by continuously engaging members to be part of the program, including as peer outreach workers, in monthly reviews of program data to address challenges, and through routine stakeholder interface meetings with law enforcement. Trust was also built through integrating services that accounted for the general health and well-being of PWID, such as mental health screenings, psychological support, paralegal support and referrals to economic empowerment, drug rehabilitation, and harm reduction programs. As a result, EpiC conducted tests twice the number of the PWID population size estimate obtained at baseline (6,032) in a context where less than half of high-risk drug users are estimated to have accessed HTS.^21^ EpiC also initiated 44% (2,659/6,032) of the baseline PWID size estimate on oral PrEP in the 2 states where, nationally, only a quarter of HIV-positive PWID are estimated to be on ART.^18^ Similarly, while the NACA estimates are from 2014 and HIV prevalence differs from state to state, we found a notably higher HIV prevalence among male PWID than that reported by NACA (8.24% vs. 2.6%, respectively).^19^ The community-based and differentiated approaches of our intervention implemented alongside PWID-centered strategies to address the structural barriers may have facilitated the project’s reach into unreached populations by building trust, which is critical for programs to bring services to the community.

Trust with the PWID community was built by continuously engaging members to be part of the program.

### Create an Enabling Environment

A notable achievement of this program was the absence of any arrests or raids of the program’s PWID clients during service delivery because of an agreement negotiated with NDLEA and other law enforcement stakeholders. This can be attributed to the strong engagement with and sensitization and training of uniformed officers, including NDLEA. NDLEA and other law enforcement groups were engaged across many forums, such as community dialogues, stakeholder interface meetings, and trainings on human rights and stigma and discrimination, together with other stakeholders to emphasize their important role in reducing barriers to access care among PWID and the public health implications of HIV among the PWID community. PWID in Nigeria are arrested multiple times in their lifetime and spend 1 or more months in prison each time,^21^ a period during which oral PrEP services are not accessible to the arrested individuals. While the EpiC program also provided HTS, TB services, and ART services to the prison population, the program was unable to provide oral PrEP due to unsupportive government policies. People in prisons in Nigeria have an estimated HIV prevalence of 2.8%, which is higher than the national estimate.^18^ To provide and sustain the provision of HIV prevention messaging and services to PWID in prison settings, greater engagement and advocacy with the government are needed.

### Plan for Additional Service Integration

While the program provided a variety of services to PWID through various channels and referrals to additional services, needle exchange and medication-assisted treatment were not directly provided. About 40% of high-risk drug users in Nigeria are reported to want drug treatment but are not able to access the services.^21^ To scale up oral PrEP services tailored to the needs of the PWID population, needle exchange and medication-assisted treatment should be provided through the same service delivery channels, including through engaging and collaborating with organizations that provide needle exchange and medication-assisted treatment.

To scale up oral PrEP services tailored to the needs of the PWID population, needle exchange and medication-assisted treatment should be provided through the same service delivery channels.

Additionally, the continuous engagement between the community, CBOs, providers, government, and law enforcement is necessary to sustain the gains in trust among the PWID community. Establishing appropriate mechanisms to allow for and facilitate this continued engagement among the various stakeholders to ensure a sustained person-centered and arrest- and stigma-free access to care for PWID are needed.

#### Integrate Feedback for Future Adaptations

While nearly all PWID (2,659 [99.92%]) who were eligible were initiated on oral PrEP, eligibility among those screened (2,661 [32.49%]) was lower than expected. The criterion “willingness to use oral PrEP as prescribed” may have contributed to the low eligibility rates. Future programs should consider documenting the frequency of reasons for ineligibility or separating “willingness” from other criteria to inform motivational counseling to enhance willingness and, thus, improve eligibility rates and subsequently, initiation rates. Increasing oral PrEP use in the PWID community in Nigeria will require continued conversations with the community to better address barriers to accessing oral PrEP services and to identify additional acceptable delivery models. In addition, improved counseling and new interventions to reach more PWID with messaging around HIV prevention and oral PrEP, including virtual interventions, should be scaled up to increase oral PrEP knowledge and address the fears and stigma specific to the PWID community.

### Focus Strategies to Reach Female PWID and Other Subpopulations

The compounded risk of arrest among female PWID compared to male PWID may be contributing to the lower proportion of HIV negative female PWID staying for screening. While only a small difference between sex was reported for ever being arrested for the possession of drugs among PWID, close to 30% of female drug users, including PWID, reported ever being arrested for sex work compared to less than 10% among all PWID and less than 5% of male drug users.^21^ This additional risk of arrest may contribute to the increased fear of arrest among female PWID and hence to the fear of accessing services or staying at the service delivery points to be screened for oral PrEP eligibility. While the program was able to prevent any arrests among PWID clients accessing services, the fear of arrest while accessing services may have remained among the PWID community. Focused interventions for reaching, reducing barriers, and improving access for more female PWID who test negative are warranted along with more female PWID-centered strategies including integration with sexual and reproductive health services.

Most of the PWID reached were aged younger than 35 years, which also warrants a need to integrate youth-friendly services, including greater online outreach and engaging more youth as peer educators and peer outreach workers in the program. Additionally, while the limited reach of older populations may be consistent with the population demographic, targeted strategies to reach older populations may be needed, such as integration with noncommunicable disease services. Greater efforts are needed to understand the needs and barriers to reaching subpopulations in this community that are harder to reach, screen, or initiate, and to better tailoring HIV services to them through DICs, outreach, and other differentiated efforts.

### Account and Adjust for Logistical and Implementation Challenges

Almost half of the implementation period coincided with COVID-19–related regional or national lockdowns and movement restrictions (March–October 2020). Additionally, a period of oral PrEP stock shortages between January and July 2020 added logistical challenges. Despite these challenges, the program was able to adjust service provision and obtain permissions to continue screening and initiating members of the PWID community on oral PrEP through multiple service delivery modalities, including online modalities, to provide continuity of care and mitigate pandemic-related barriers to accessing services.

## CONCLUSION

Additional HIV prevention interventions and services are needed to control the HIV epidemic among the PWID community, and the provision of oral PrEP is one feasible option. We demonstrated that, even with the logistical challenges brought on by COVID-19, commodity stock-outs, and the challenge of serving a harder-to-reach PWID community, provision of oral PrEP is possible through community-led, differentiated, and person-centered service and should be replicated, scaled up, and evaluated by other HIV programs serving PWID.

While this program was implemented in only 2 of the 36 states in Nigeria, the 2 states are in different geopolitical zones with different geographic and political contexts. However, the PWID communities in the 2 states face similar challenges while accessing services. The legal barriers and the stigma and discrimination associated with being members of the PWID community are also similar in the 2 states. Our implementation experience and the lessons learned in the 2 states were similar and hence could likely be generalized to other areas of the country.

Our implementation experience and the lessons learned in the 2 states were similar and hence could likely be generalized to other areas of the country.

Optimizing the impact of a biomedical prevention intervention, like oral PrEP, with marginalized populations requires not only making the product available but also investing efforts to tailor the intervention and create an enabling, trusting environment in which members of that community feel safe and motivated to access services. Future PrEP programs for the PWID community, especially those in contexts where injection drug use is criminalized, should incorporate strategies to address structural barriers, including the engagement of law enforcement. Integration of oral PrEP and other HIV services into family planning services, noncommunicable disease services, harm reduction and medication-assisted treatment, and services in prison should also be strongly considered to tailor health services to better meet the needs of the PWID community and its harder-to-reach subpopulations.

To sustain this oral PrEP service delivery model for PWID, it is necessary to advocate for a mechanism of collaboration and coordination between the health and law enforcement departments and for the adoption of the differentiated and peer-led service delivery model by the government. It is also important to sensitize and build the capacity of service providers within public health facilities to ensure they provide stigma-free services to PWID, thus ensuring their integration into the public health system. Other funding sources, including direct government funding of the program, must be explored. Research and cost-effectiveness evaluations are needed to understand the model’s contribution to reducing HIV transmission among the PWID community and hence to national HIV epidemic control efforts.

Finally, the ongoing efforts at achieving HIV epidemic control will remain a mirage if we are unable to scale up oral PrEP services and ensure all individuals who need the services can access them. For example, men in general have been left out of many oral PrEP programs and our experience highlights a program that reached men with the needed HIV prevention services. Specific messages that target men and other subpopulations should be part of routine demand-creation efforts across all settings, and more differentiated service delivery models that cater to the needs of these different populations and groups are needed. The lessons from our program could also become valuable when new HIV prevention interventions, including long-acting cabotegravir and dapivirine vaginal ring, are to be introduced as part of a combination HIV prevention package.

## Notes

**Peer Reviewed**

**First published online:** February 10, 2023.
